# Transparent Thin-Film Transistors Based on Sputtered Electric Double Layer

**DOI:** 10.3390/ma10040429

**Published:** 2017-04-20

**Authors:** Wensi Cai, Xiaochen Ma, Jiawei Zhang, Aimin Song

**Affiliations:** School of Electrical and Electronic Engineering, University of Manchester, Manchester M13 9PL, UK; wensi.cai@postgrad.manchester.ac.uk (W.C.); xiaochenma0531@gmail.com (X.M.); jiawei.zhang@manchester.ac.uk (J.Z.)

**Keywords:** electric-double-layer (EDL), radio frequency (RF) magnetron sputtered SiO_2_, transparent thin-film transistors (TFTs)

## Abstract

Electric-double-layer (EDL) thin-film transistors (TFTs) have attracted much attention due to their low operation voltages. Recently, EDL TFTs gated with radio frequency (RF) magnetron sputtered SiO_2_ have been developed which is compatible to large-area electronics fabrication. In this work, fully transparent Indium-Gallium-Zinc-Oxide-based EDL TFTs on glass substrates have been fabricated at room temperature for the first time. A maximum transmittance of about 80% has been achieved in the visible light range. The transparent TFTs show a low operation voltage of 1.5 V due to the large EDL capacitance (0.3 µF/cm^2^ at 20 Hz). The devices exhibit a good performance with a low subthreshold swing of 130 mV/dec and a high on-off ratio > 10^5^. Several tests have also been done to investigate the influences of light irradiation and bias stress. Our results suggest that such transistors might have potential applications in battery-powered transparent electron devices.

## 1. Introduction

Oxide semiconductors have gained much attention particularly in the last decade due to their high mobility, good uniformity over large area, low fabrication temperature, and low cost [1,2,3]. Nowadays, transparent oxide-based thin-film transistors (TFTs) have been used in many applications such as active-matrix liquid crystal displays and organic light-emitting diode displays [4,5]. However, by using conventional dielectrics, such TFTs usually require large operation voltages, resulting in high power consumption and thus making them unsuitable for battery-powered portable applications.

In order to lower the operation voltage, the most common method is using a thin dielectric layer, like ultrathin cross-linked films or self-assembled monolayers [6,7,8]. However, this might cause a large gate leakage because of the challenges of maintaining a very high degree of uniformity over a large area. Another way is using high-κ dielectrics to increase the gate coupling between the electrode and the channel layer [9,10,11]. In 2015, Zhou and coworkers reported that, by using high-κ Pb(Zr_0.52_Ti_0.48_)O_3_ (PZT) as dielectric layer, the operation voltage of the devices could be reduced to 1 V [10]. However, such high-κ dielectric materials usually have a problem of threshold voltage control due to the large fixed charge trap densities and therefore the devices might have a serious issue of long-term reliability [12].

Recently, many groups have tried using polymer electrolytes (polyelectrolytes) or ionic liquids (ion gels) as gate dielectric to reduce the operation voltage due to the large electric-double-layer (EDL) capacitance formed at the channel/dielectric interface [13,14,15]. Pu et al. reported on MoS_2_-based EDL TFTs using ion gel as gate dielectric in 2012 and achieved an operation voltage less than 1 V [16]. However, the chemical stability of polymer electrolytes is not good, especially at high temperatures. Ionic liquids or ion gels also have limitations due to the difficulties of controlling their shapes, uniformity, and thickness. Some solid-state electrolytes, mainly SiO_2_ and Al_2_O_3_, were therefore introduced [17,18,19,20,21]. Jiang et al. reported transparent EDL-based TFTs that could reach an operation voltage of 1 V and a current on-off ratio larger than 10^6^ by using plasma-enhanced chemical vapor deposition (PECVD) deposited SiO_2_ as a dielectric layer [22]. However, the processing gases and the by-products of PECVD might be toxic, flammable, and explosive.

In manufacturing, sputtering techniques offer a more cost-effective way for the fabrication of large area oxide-semiconductor based electronics. Recently, we developed a novel way to produce SiO_2_ electrolyte using sputtering method and the obtained TFTs showed an 1 V operation voltage and a subthreshold swing close to the theoretical limit [23]. The devices however were fabricated using non-transparent source-drain and gate electrodes.

In this paper, we report transparent EDL-based TFTs that are fabricated at room temperature with radio-frequency (RF) magnetron sputtering method only, which is, to the best of our knowledge, the first all sputtered, transparent, EDL transistor to date. The transistors have an operation voltage of 1.5 V and a maximum transmittance about 80% in the visible light region. Also, the transistors show good electrical properties with a current on-off ratio larger than 10^5^ and a subthreshold swing about 130 mV/dec.

## 2. Materials and Methods

**Figure 1:**
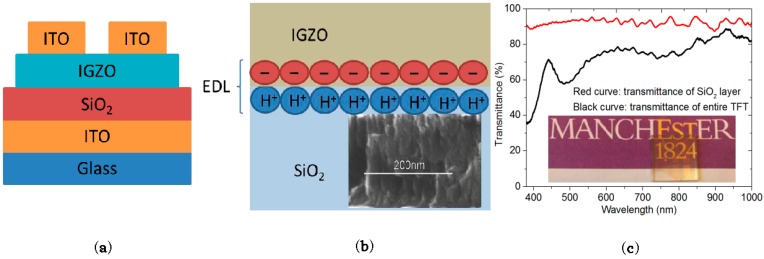
(**a**) Schematic diagram of transparent EDL transistors; (**b**) Schematic cross-sectional view of an EDL TFT when applying a positive gate voltage. Inset: cross-sectional scanning electron microscope view of the gate dielectric layer; (**c**) Optical transmission spectra of the thickest part in the TFT (black curve) and SiO_2_ layer (red curve). Inset: a photo of the transparent TFT on glass substrate.

Transparent Indium-Gallium-Zinc-Oxide (IGZO)-based TFTs were fabricated on glass substrates at room temperature using bottom gate top contact structure. A schematic diagram of the transistors is shown in Figure 1a. First, 200-nm-thick ITO was deposited on as the gate electrode by RF magnetron sputtering using indium tin oxide (ITO) target (90 wt % In_2_O_3_ and 10 wt % SnO_2_) at 40 W. Then, a 200-nm-thick SiO_2_ gate dielectric layer was deposited on by RF magnetron sputtering using SiO_2_ target at 85 W. After that, a 70-nm-thick IGZO as the channel layer was deposited on by RF magnetron sputtering at 40 W. Finally, 200-nm-thick ITO source and drain electrodes were deposited on by RF magnetron sputtering using ITO target at 40 W. The sputtering processes were kept at a pressure of 5 × 10^−3^ mbar in pure argon. All layers were patterned using shadow masks. The channel length and width were 60 µm and 2 mm, respectively. The electrical properties of the devices were measured using Agilent E5270B semiconductor analyzer (Agilent, Santa Clara, CA, USA) and Agilent E4980A LCR metre (Agilent) in air ambient. The cross-sectional view of SiO_2_ was investigated by using FEI Nova NanoSEM 450 scanning electron microscope (SEM, FEI, Hillsboro, OR, USA).

## 3. Results and Discussion

Figure 1a shows a schematic diagram of the devices. The inset of Figure 1b shows a cross-sectional SEM image of SiO_2_ gate dielectric layer, indicating a porous microstructure (full image can be found in Supplementary Figure S1). As such, our previous study indicates that the SiO_2_ layer might absorb some water molecules from the ambient air [23]. When a positive bias is applied to the gate electrode, the ionized protons inside the gate electrolyte are accumulated close to the dielectric/channel interface due to the electric field and thus induce electrons inside the IGZO channel layer to accumulate at channel/dielectric interface, which behaves like a normal EDL, as shown in Figure 1b.

Figure 1c shows the optical transmission spectra of the thickest part in the TFT and a 200-nm-thick sputtered SiO_2_ layer on glass from 380 to 1000 nm. The maximum transmittance of the TFTs in the visible spectrum (400–800 nm) is about 80%, indicating that the TFTs are transparent to visible light. This can be further demonstrated by the inset of Figure 1c, as the background graph can be seen clearly through the TFT arrays. The drop in the transmittance between 450 nm and 550 nm is caused by the interference of multiple reflections in the transparent films [24,25,26,27].

**Figure 2:**
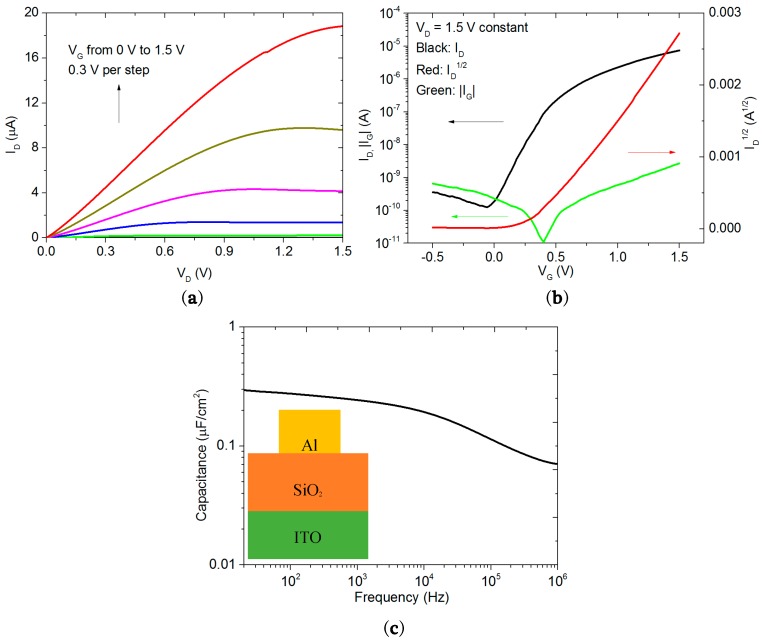
Electrical properties of the TFTs (**a**) Output characteristics with *V*_D_ swept from 0 V to 1.5 V and *V*_G_ swept from 0 V to 1.5 V with a step of 0.3 V; (**b**) Drain current, gate leakage current and square root of drain current with *V*_G_ swept from −0.5 V to 1.5 V at *V*_D_ = 1.5 V; (**c**) Capacitance of SiO_2_ gate dielectric as a function of frequency (20 Hz to 1 MHz) with inset showing the capacitance test structure. The channel length and width used for the devices are 60 µm and 2 mm, respectively. The SiO_2_ thickness used for capacitance measurement is 200 nm.

Figure 2a shows the output characteristics of the transparent TFTs with the gate voltage *V*_G_ varying from 0 V to 1.5 V in a step of 0.3 V, indicating the devices operate in N-type enhanced mode. It shows a good linear region at low drain voltages, *V*_D_, indicating a low resistance of the source/drain contacts. A good saturation region is also obtained at large *V*_D_ and a drain current, *I*_D_, of 19 µA is obtained under a bias condition of *V*_D_ = 1.5 V and *V*_G_ = 1.5 V. The decrease of *I*_D_ in the saturation regime may be attributed to the charge trapping effect at the channel/dielectric interface [28]. Figure 2b shows the corresponding transfer characteristics of the devices measured at a 1.5 V-fixed drain voltage. The gate leakage current, *I*_G_, is found to be about 2.6 nA at *V*_G_ = 1.5 V, which is far less than the *I*_D_. The TFT exhibits a good performance with a large current on-off ratio > 10^5^, a low subthreshold swing about 130 mV/dec, and a turn-on voltage around 0 V. From *I*_D_^1/2^ as a function of *V*_G_ shown in Figure 2b, the threshold voltage is calculated to be about 0.4 V by using the x-axis intercept of the curve.

Figure 2c shows the specific gate capacitance of SiO_2_ electrolyte in the frequency range between 20 Hz and 1 MHz using ITO/SiO_2_/Al test structure, as shown in the inset of Figure 2c. For conventional SiO_2_, which has a dielectric constant of 3.9, a 200-nm-thick SiO_2_ insulator layer has a specific capacitance of ~17.3 nF/cm^2^, which is independent of frequency. In Figure 2c, it is found that the capacitance of the sputtered SiO_2_ electrolyte decreases with increasing frequency. At 20 Hz, the specific capacitance is 300 nF/cm^2^ and at 1 MHz, it drops to 70 nF/cm^2^. The high capacitance is probably due to the mobile protons in the SiO_2_ electrolyte [23,29]. Three coordinate oxygen centres, Si-OH^+^-Si, are formed in the SiO_2_ layer due to the absorption of H_2_O molecules [17,30,31]. Since the bonds between oxygen and hydrogen are not stable, the external electric field will draw the proton from the hydroxyl group to the neighbouring ones. When applying a positive bias, the protons will accumulate at the dielectric/channel interface, forming a thin boundary layer, and induce electrons in the channel. Such process is similar to the formation mechanism of EDL in transistors gated by ionic liquids or ion gels [13,32]. As the protons in the SiO_2_ electrolyte have a low ionic mobility, at high frequencies, the protons do not have enough time to move to the interface [29]. This results in the frequency-dependent capacitance shown in Figure 2c. In this experiment, the large EDL gate capacitance enables the low operation voltage of the IGZO TFTs.

The mobility (${µ}_{FE}$) in the saturation region can be derived from the following equation:

(1) $$I_{\mathrm{DS}}=\left(\frac{W}{2L}{µ}_{FE}C\right){\left(V_{GS}-V_{TH}\right)}^{2}$$

where *W* is the channel width, *L* is the channel length, and *C* is the capacitance per unit area. By using the capacitance value at 100 Hz, the estimated mobility is ~3 cm^2^/Vs. As the devices use a 70-nm-thick channel layer without any post-treatment, this mobility value is considered to be reasonable and can be further improved by thermal annealing or reducing the channel thickness [33,34,35].

**Figure 3:**
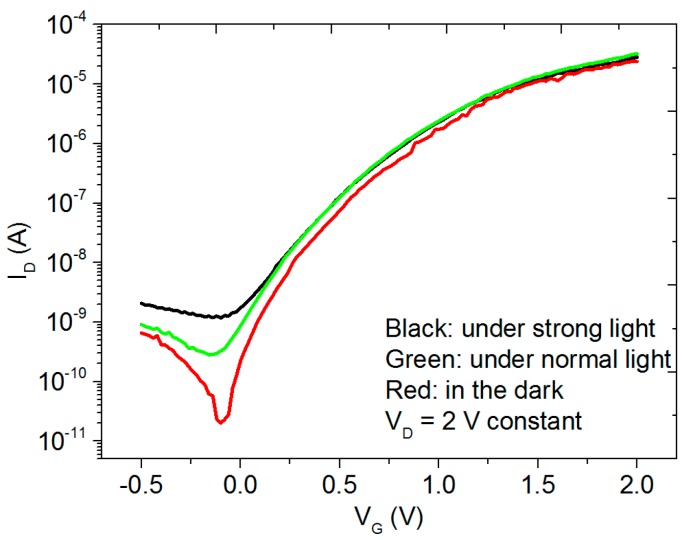
Electrical properties of the transistors under different light conditions when *V*_G_ swept from −0.5 V to 2 V at *V*_D_ = 2 V. Black curve: under strong light (around 2000 lx giving by a white light LED three centimetres away from the sample). Green curve: under normal room light (around 300 lx). Red curve: in the dark. The channel length and width used for the devices are 60 µm and 2 mm, respectively.

For the transparent TFTs, it is important to investigate the influence of light irradiation on the electrical performance. In order to make the change more obvious, a 2-V fixed drain voltage has been used for the tests under different light conditions and the obtained transfer characteristics are shown in Figure 3. Off current increases clearly when increasing the light, which changes from 0.02 nA in the dark to 0.28 nA under normal room light (around 300 lx) and further to 1.5 nA under strong light (around 2000 lx) with a white light LED about 3 cm away from the device, resulting in a decrease of current on-off ratio. The threshold voltage also shifts towards negative direction after exposure to light. The light stress stability under around 2000 lx white light has also been tested as shown in Supplementary Figure S2. The subthreshold swing remains almost the same but the threshold voltage decreases around 0.1 V after six hours. There might be two reasons for the shift in *V*_TH_. Firstly, light can induce extra free electrons in the IGZO layer [36]. As IGZO is reported to be sensitive to the ambient environment, the other reason might be the adsorption or desorption of illumination induced O_2_ molecules in the IGZO layer [37,38]. Some O_2_ molecules might be pre-absorbed and react with the conducting electrons in the channel layer to form O_2_^−^. Under light illumination, the holes generated from the light induced electron-hole pairs might react with these O_2_^−^ causing desorption of O_2_ and resulting in the decrease of threshold voltage.

After switching off the strong light, the sample has then been measured in the dark and the transfer characteristics change back to the initial values shown as the red curve in Figure 3. Such behavior indicates that the changes induced by light are reversible.

**Figure 4:**
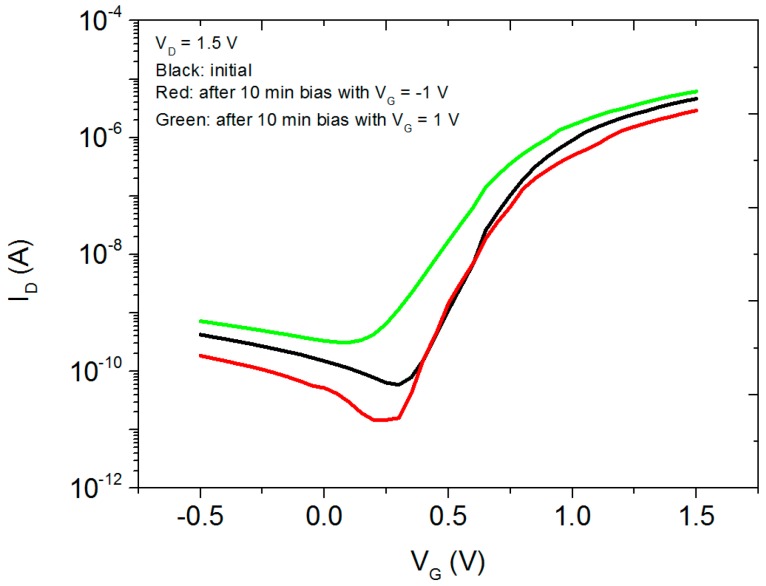
Bias stress testing. Transfer characteristics of the TFT with *V*_D_ = 1.5 V at different bias stress voltages. Black curve: initial transfer curve. Red curve: transfer curve after 10 minutes’ constant bias at *V*_G_ = −1 V. Green curve: transfer curve after 10 minutes’ constant bias at *V*_G_ = 1 V. The channel length and width used for the devices are 60 µm and 2 mm, respectively.

In order to investigate the stability of the transparent EDL TFTs, both positive and negative bias stress tests are carried out by applying constant gate biases at 1 V for positive bias stress test and −1 V for negative bias stress test. Figure 4 shows the transfer characteristics of the devices before and after bias stresses. The subthreshold swing remains almost the same after negative bias and increases to 0.2 V/dec after positive bias. The threshold voltage shows a slight right shift, +0.01 V, after negative bias and a left shift, −0.12 V, after positive bias. These changes are typical for SiO_2_-based EDL transistors [39,40]. When the gate is positively biased, the protons inside the gate electrolyte continuously accumulate at the dielectric/channel interface due to the applied electric field and thus induce the electrons inside the channel layer to accumulate at channel/dielectric interface, which results in a decrease of threshold voltage and an increase of on current. When the gate is negatively biased, the protons inside the dielectric are repelled to the dielectric/gate interface. Therefore, the number of electrons accumulating at channel/dielectric interface decreases. This results in a right shift of threshold voltage and a decrease of on current. After removing the bias and leaving the devices in air for 10 minutes, the transfer characteristics of the device return to its initial condition, which means the change induced by bias stress is reversible. By using a capping layer, it is possible that the stability of our devices can be further improved [41].

## 4. Conclusions

In conclusion, we report, to the best of our knowledge, the first all-sputtered, transparent EDL TFTs fabricated at room temperature. The operation voltage is low due to the large specific EDL capacitance formed by the sputtered-SiO_2_ solid gate dielectric. The TFTs exhibit good electrical properties with a current on-off ratio > 10^5^ and a subthreshold swing of about 130 mV/dec. A maximum transmittance of about 80% is also observed in the visible light range. Considering the low operation voltage and transparent features of the EDL TFTs, our results demonstrate the potential in transparent electronics and low power circuit applications. Also, as the entire transistors are deposited only by RF magnetron sputtering, the method is compatible with large-area electronics manufacturing.

## Supplementary Materials

The following are available online at www.mdpi.com/1996-1944/10/4/429/s1. Figure S1: Cross-sectional SEM image of the sputtered SiO_2_ electrolyte. Figure S2: (a) Transfer characteristics; (b) threshold voltage and subthreshold swing of the TFT after white-light stress (around 2000 lx) for several durations.
