# Supplementary material for: Transparent Thin-Film Transistors Based on Sputtered Electric Double Layer
Source: Materials (Basel). 2017 Apr 20;10(4):429. doi: 10.3390/ma10040429 (PMC5506904; doi:10.3390/ma10040429)
Supplement: Supplementary file 1 [file materials-10-00429-s001.pdf]

Supplementary Materials: Transparent Thin-Film Transistors Based on Sputtered Electric Double Layer

Wensi Cai, Xiaochen Ma, Jiawei Zhang, and Aimin Song

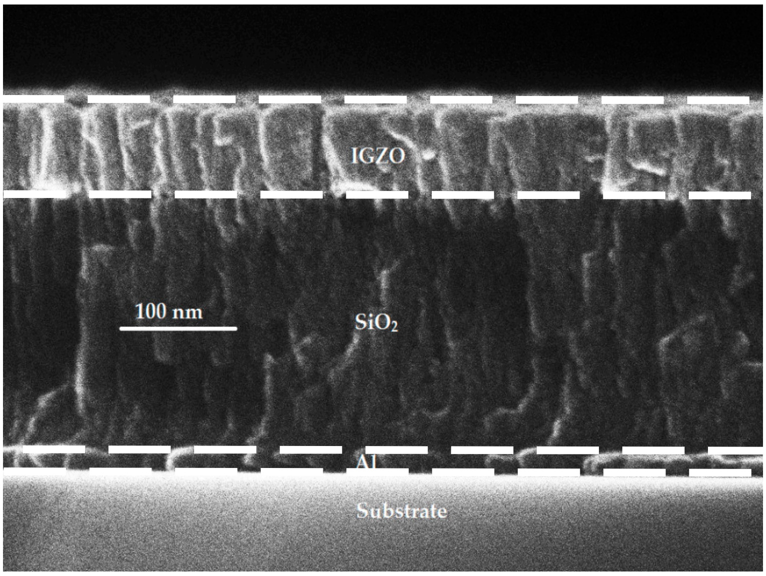

Figure S1. Cross-sectional SEM image of the sputtered SiO_2 electrolyte.

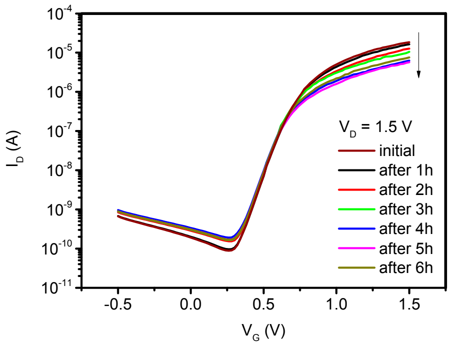

(**a**)

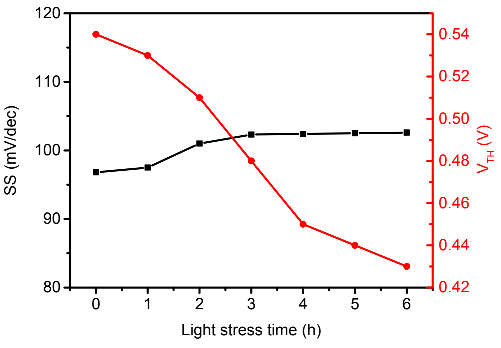

(**b**)

Figure S2. (a) Transfer characteristics, (b) threshold voltage and subthreshold swing of the TFT after white-light stress (around 2000 lx) for several durations.
